# Fluoridated orthodontic adhesives: Implications of release and recharge and their impact on shear bond strength in demineralized tooth surfaces

**DOI:** 10.34172/joddd.2023.40594

**Published:** 2023-11-11

**Authors:** Mohammed Sabah Yaseen, Neam F Agha, Raya Jasim

**Affiliations:** Department of Pedodontics, Orthodontics and Preventive Dentistry, College of the Dentistry, University of Mosul, Mosul, Iraq

**Keywords:** Adhesive, Demineralized teeth, Fluoride release, Recharge

## Abstract

**Background.:**

This study measured fluoride release from a light-cured orthodontic adhesive resin (Vega type) at three time intervals (one day, one week, and one month), investigated the rechargeability of the resin, and assessed its impact on shear bond strength in demineralized tooth surfaces.

**Methods.:**

This study used 30 recently extracted upper premolar teeth to explore the effects of fluoride release over specific time intervals. The teeth underwent demineralization and were categorized into groups based on time intervals: one day, one week, and one month. Subgroups within each interval underwent fluoride recharging through fluoride varnish application. Fluoride release and shear bond strength were assessed after etching with phosphoric acid gel, applying the orthodontic adhesive, and curing. The samples were stored in deionized water. Fluoride quantification used a selective electrode, while shear bond strength assessment employed a universal testing machine. Finally, statistical analysis of the data was performed using SPSS 22.

**Results.:**

The study found that after one month, the adhesive had the highest fluoride release and shear bond strength mean values. There were significant differences in fluoride release and shear bond strength between the various groups studied.

**Conclusion.:**

The application of fluoride varnish around the orthodontic bracket resulted in a positive effect on the shear bond strength of the bracket.

## Introduction

 Among the diverse array of orthodontic interventions, multi-bracket treatment persists as the preeminent modality extensively employed within the realm of orthodontic applications. While it offers several distinct advantages, it is not devoid of inherent risks, the most encountered being bracket detachment or the onset of gingival inflammation. Furthermore, this treatment approach renders patients more susceptible to caries, decalcification, and enamel demineralization, accentuating the importance of comprehensive and meticulous oral care during treatment.^1^

 The occurrence of enamel demineralization, commonly referred to as white spot lesions (WSLs), represents a formidable risk factor intrinsic to orthodontic treatment. Particularly noteworthy is the heightened susceptibility observed in cases where oral hygiene is compromised, accompanied by protracted plaque accumulation in the vicinity of brackets. Given this context, safeguarding the integrity of the enamel surface poses a formidable challenge for orthodontic practitioners, necessitating their unwavering commitment to meticulous preventive measures and diligent patient education.^2^

 Enamel demineralization, manifesting as WSLs, signifies the incipient stage of carious lesion formation. Consequently, the effective prevention of WSL occurrence has emerged as a highly prominent research area. Notably, scientific literature has reported that approximately one-third of WSLs can be arrested before progressing into overt caries. As a result, the utilization of agents with caries-preventive properties or those capable of halting primary lesions is strongly recommended for orthodontic patients.^3,4^

 The main approach for preventing the formation of WSLs lies in patient education and fostering motivation concerning oral hygiene practices. Additionally, various other methods have proven effective in this endeavor, including fluoride gels, varnishes, toothpaste, and mouth rinses. Moreover, incorporating fluoride into orthodontic adhesives has emerged as a widely adopted technique in combating WSLs.^2^

 Fluoride-releasing materials can absorb fluoride ions from the oral environment, replenishing the lost fluoride content. This phenomenon, known as fluoride recharge, plays a pivotal role in enabling these materials to exert a prolonged inhibitory effect on enamel demineralization.^5^

 In conjunction with the emergence of WSLs, the issue of bracket detachment emerges as a significant concern inherent to the landscape of multi-bracket orthodontic therapy. Bracket detachment constitutes a matter of considerable gravity, particularly in select clinical contexts, given its capacity to exert a substantial influence on the overarching efficacy of treatment regimens and their temporal dimensions. Scholarly literature has meticulously chronicled notable instances of bracket detachment, mandating comprehensive scrutiny owing to its conceivable ramifications for the ultimate outcomes of orthodontic interventions.^1^

 The success of bracket bonding in orthodontic treatment relies on achieving shear bond strengths (SBS) within a range of 4‒10 MPa. This range ensures that the bonded brackets can withstand the orthodontic forces and masticatory pressures encountered during treatment. SBS values < 4 MPa may lead to bracket detachment, compromising treatment outcomes, while values exceeding 10 MPa can pose challenges during bracket removal. Striking a balance within this SBS range allows for effective force transmission, controlled tooth movement, and overall treatment success. Thus, meticulous attention is given to bonding protocols to attain optimal SBS values, ensuring both the stability of brackets during treatment and their safe removal at the end of treatment.^6^

 The influence of fluoride application on the bond strength is a subject of investigation. Emerging research indicates that the temporal aspect of fluoride application, whether preceding or succeeding bracket placement, holds significance in determining its impact on bonding strength.^7,8^

## Methods

###  Sample collection and inclusion criteria

 The present study used recently extracted upper premolar teeth in private clinics and dental centers in Mosul City. The selection of teeth met the inclusion criteria, such as normal size, intact buccal surfaces without cracks or fractures resulting from extraction, a healthy dental structure without caries or restorations, and no prior orthodontic treatment. The samples were cleansed and stored in daily-changed distilled water at room temperature to prevent bacterial growth.

###  Grouping of the samples

 Thirty samples were divided into three main groups to study fluoride release over specific intervals: one day, one week, and one month. Each group consisted of ten samples. Within each group, two subgroups of five samples were formed. One subgroup received fluoride recharge through fluoride varnish application, while the other subgroup did not undergo recharge treatment. Fluoride release and SBS were then assessed for all the samples accordingly.

###  Demineralization protocol

**Figure 1:**
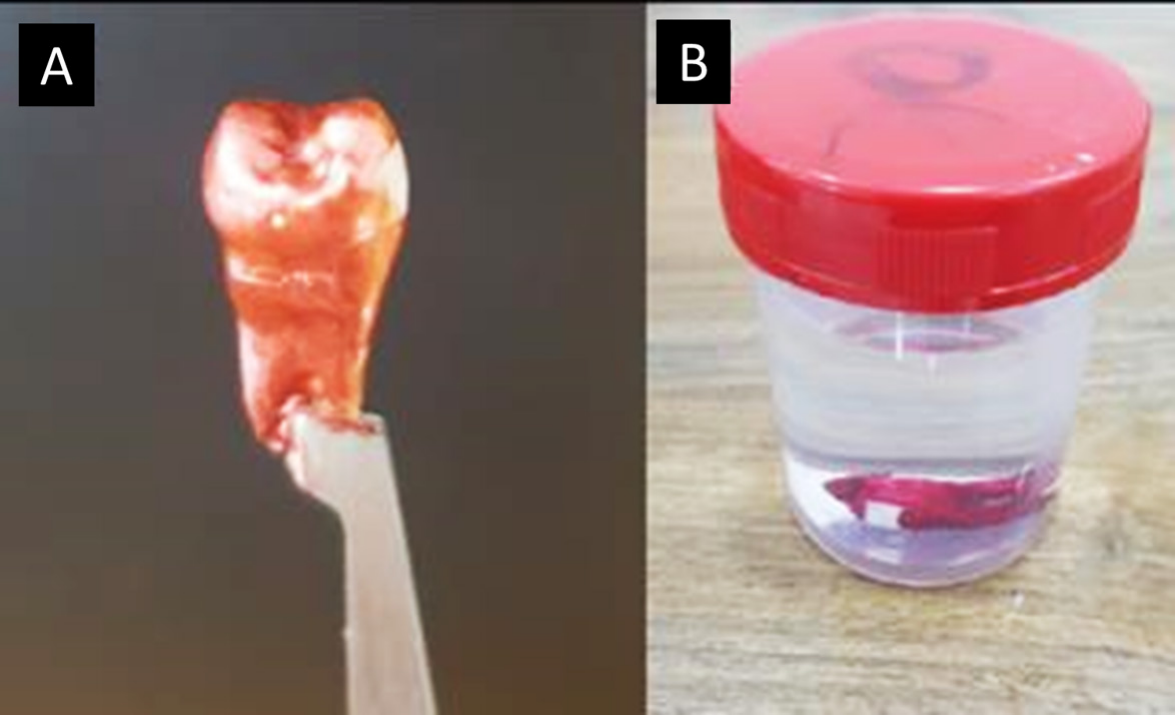
A: The sample after nail varnish was applied. B: The sample immersed in demineralization solution

 For demineralization, acid-resistant varnish (nail varnish) was applied to protect the tooth, leaving a specific 3 × 4-mm window of exposed enamel on the middle third of the buccal surface of the crown, which allowed targeted acid attack solely on the exposed enamel, while the rest of the tooth remained protected by the acid-resistant varnish layer (Figure 1A).^9^

 Each tooth was individually submerged in a demineralization solution comprising 2.2-mM CaCl_2_, 2.2-mM KH_2_PO_4_, and 0.05-M acetic acid to induce artificial carious lesions. The solution was adjusted to a pH of 4.4 using 1-M KOH using a Eutech 700 pH Meter. Submersion took place at a temperature of 37°C for 96 hours. This controlled immersion period allowed for the development of initial artificial carious lesions within the tooth structures (Figure 1B).^10^

###  The sample preparation

 The samples were mounted on plastic polyvinyl chloride (PVC) rings with specific dimensions (a 20-mm outside diameter, an 18-mm inside diameter, and a 30-mm height). The mounting process involved several steps to ensure precise positioning. Firstly, the PVC rings were partially filled with dental stone, stopping at around half their height. The tooth samples were then secured onto the stone surface using soft, sticky wax to center them within the plastic ring and position them perpendicular to the base. The sample assembly was placed on a glass slab attached to a dental surveyor to maintain consistency and alignment. The long axis of each tooth was aligned parallel to the analyzing rod of the surveyor, replicating the direction of force application during the SBS test.^11^

 Finally, the PVC rings were filled with autopolymerizing cold-cured acrylic resin until it reached the cementoenamel junction (CEJ) level to stabilize and secure the tooth samples for accurate analysis and testing.

###  Bonding procedure

 The mounted samples underwent a meticulous polishing procedure using fluoride-free pumice and a rubber prophylactic cup. The buccal surface of each tooth was then etched with a 37% phosphoric acid gel for 30 seconds, followed by rinsing and drying. The entire sample assembly was positioned on an articulator with a prefabricated base for stability and alignment. Subsequently, stainless-steel metallic brackets of the standard edgewise type (Dentaurum, Ispringen, Germany), were securely held using clamping tweezers, and Vega Ortho UV light-cured orthodontic adhesive resin (DFL, Brazil) was evenly applied to the bracket’s base. Careful positioning of the bracket at the center of the buccal surface of the premolar tooth, maintaining a 4-mm distance from the occlusal surface, was ensured using the Boon’s gauge as a guide for accurate placement.

**Figure 2:**
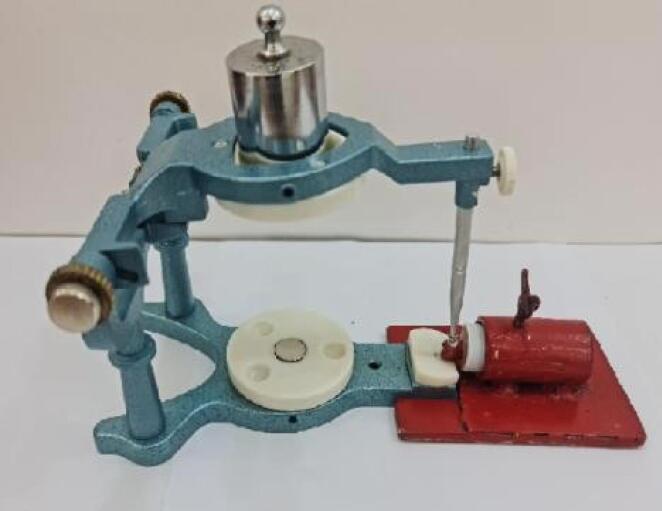
The whole sample was positioned on the articulator using a prefabricated base

 A standardized load of 200 gm was applied perpendicular to the bracket slot on the articulator arm, as seen in Figure 2, to ensure uniform resin thickness without air voids.^12^ The excess resin was removed, and curing was performed using an LED light-curing device with a wavelength range of 385‒515 nm and an illumination intensity of 1200‒1500 MW/cm^2^. The curing light was calibrated for every 5 samples using a radiometer. The curing process involved 20 seconds of light exposure on the mesial and distal sides of the bracket, with the tip positioned 2 mm away from each edge.^13^ Finally, the specimens were placed in a container with 5 mL of deionized water.

###  Fluoride recharging

 Each sample was thoroughly dried in the recharging groups and then subjected to a topical fluoride varnish (FluoroDose, USA) in one application using a brush applicator around the bracket. After a 3-minute interval, the samples were lightly moistened with a mild air/water spray and individually stored in 5 mL of deionized water until further analysis.^14^

###  Fluoride analysis

**Figure 3:**
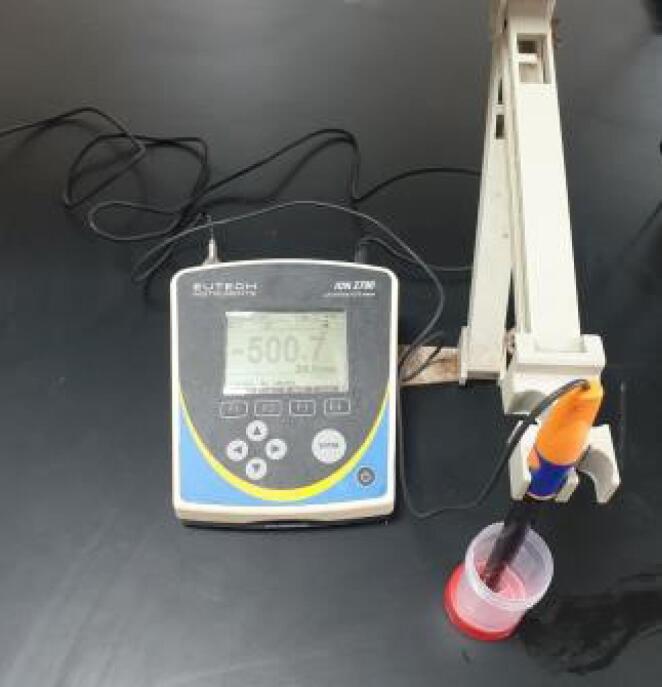
Fluoride analysis

 Fluoride analysis took place at the central laboratory in the College of Agriculture and Forestry, Mosul University. Total solubilized fluoride was determined using a fluoride-selective electrode, with readings (in mV) recorded once stabilization occurred, as shown in Figure 3. To ensure accuracy, the electrode was pre-calibrated using a series of fluoride calibration solutions, creating a calibration curve that facilitated the conversion of millivolt readings to fluoride concentrations expressed in parts per million (ppm). This process allowed precise quantification of fluoride levels in the samples and enabled meaningful conclusions about their fluoride content.

###  Measuring shear bond strength

 The SBS test was performed at the Operative Department laboratories in the College of Dentistry, Mosul University, utilizing a universal testing machine (Gester, China) with a crosshead speed of 0.5 mm/min. A knife edge blade was positioned to exert force on the tooth-bracket interface in an occlusogingival direction. The necessary load to debond or initiate bracket failure was recorded in Newton units and then converted to megapascals (MPa) by dividing the failure load into Newton units by the standardized base surface area of the brackets, which was 10.03 mm^2^ in this study as per the manufacturer’s specifications. The steps were repeated using newly extracted teeth with fluoridated adhesive to measure fluoride release and recharging at different time intervals. Fluoride release was quantified with a fluoride-selective electrode, and SBS was assessed using a universal testing machine.

###  Statistical analysis

 Statistical analysis was performed using SPSS 22. The normal distribution of variables was checked using the Shapiro-Wilk test. The analysis included the following procedures:

1. Descriptive statistics: Mean, standard deviation, range, minimum, and maximum values, and standard errors of the mean values were calculated for each variable.
2. One-way analysis of variance (ANOVA) was used to detect significant differences between the groups regarding fluoride release and SBS, with the significance level set at *P* ≤ 0.05.
3. Duncan’s multiple range test was conducted as a post hoc test to determine significant differences in fluoride release and SBS between the groups. Significance was considered at *P* ≤ 0.05.

## Results

###  Fluoride release results

####  Descriptive analysis of fluoride release

 The analysis of the study findings revealed that the month-after group had the highest mean value. In contrast, the week-before group exhibited the lowest mean value among all the groups. The minimum value was observed in the week-before group, whereas the maximum value was observed in the month-after group (Table 1).

**Table 1:** Descriptive statistics for the fluoride release

**Time**	**Variables**	**Mean**	**SD**	**Minimum**	**Maximum**
Day	Before	0.379	0.0221	0.33	0.41
	After	0.957	0.0186	0.94	0.99
Week	Before	0.096	0.0394	0.03	0.15
	After	0.685	0.0321	0.64	0.73
Month	Before	0.212	0.1336	0.08	0.48
	After	1.087	0.0577	1.00	1.14

The measurements in ppm.

####  Analysis of variance of fluoride release

 The results of the ANOVA statistical test are presented in Table 2, indicating a significant difference (*P* ≤ 0.05) between the mean values of the fluoride release in the present study.

**Table 2:** ANOVA for the mean values of fluoride release

	**Sum of squares**	***df***	**Mean square**	**F**	**Sig.**
Between groups	5.452	5	1.090	214.770	0.000
Within groups	0.198	39	0.005		
Total	5.650	44			

*df*: degree of freedom; F: F test; Sig: is significant level at (*P* ≤ 0.05).

 The results of Duncan’s multiple range test are illustrated in Table 3, revealing that all the groups in the fluoride release test showed a significant difference when compared to each other.

**Table 3:** Duncan’s multiple-range test of fluoride release

**Time**	**Variables**	**Mean**	**SD**	**Duncan**
Day	Before	0.379	0.0221	C
	After	0.957	0.0186	E
Week	Before	0.096	0.0394	A
	After	0.685	0.0321	D
Month	Before	0.212	0.1336	B
	After	1.087	0.0577	F

Different letters mean significant difference at (*P* ≤ 0.05).

###  Shear bond strength results

####  Descriptive analysis of shear bond strength 

 The analysis of the findings from this study revealed that the month-after group exhibited the highest mean value, whereas the day-before group displayed the lowest mean value among all the groups. The day-before group demonstrated the minimum value, while the month-after group exhibited the maximum value (Table 4).

**Table 4:** Descriptive statistics for the shear bond strength

**Time**	**Variables**	**Mean**	**SD**	**Minimum**	**Maximum**
Day	Before	3.283	0.908	2.30	4.38
	After	5.242	0.898	3.69	5.96
Week	Before	5.848	1.787	3.81	7.65
	After	7.632	1.424	5.18	8.82
Month	Before	6.122	2.491	3.57	9.77
	After	7.657	1.387	6.40	10.02

The measurements in MPa.

####  Analysis of variance of shear bond strength

 The results of the ANOVA statistical test are presented in Table 5, indicating a significant difference (*P* ≤ 0.05) between the mean values of the SBS in the present study.

**Table 5:** ANOVA for the mean values of shear bond strength

	**Sum of squares**	***df***	**Mean square**	**F**	**Sig.**
Between groups	66.983	5	13.397	5.363	0.002
Within groups	59.952	24	2.498		
Total	126.934	29			

*df*: degree of freedom; F: F test; Sig: is significant level at (*P* ≤ 0.05)

 The results of Duncan’s multiple range test are illustrated in Table 6, which shows that the day-before group significantly differed with other groups except the same group after recharge. The week-before group significantly differed from only the day-before group.

**Table 6:** Duncan’s multiple range test of shear bond strength

**Time**	**Variables**	**Mean**	**SD**	**Duncan**
Day	Before	3.283	0.908	A
	After	5.242	0.898	AB
Week	Before	5.848	1.787	BC
	After	7.657	1.424	C
Month	Before	6.122	2.491	BC
	After	7.632	1.387	C

Different letters mean significant difference at (P ≤ 0.05).

## Discussion

 Orthodontic patients frequently face a heightened susceptibility to dental caries throughout orthodontic treatment, particularly when they exhibit suboptimal adherence to oral hygiene instructions. Enamel demineralization represents an undesirable yet commonly encountered complication arising from orthodontic fixed appliance therapy. The challenge of effectively managing dental plaque both before and during fixed orthodontic treatment while simultaneously preserving the bond strength of brackets has consistently been a subject of research in orthodontics.

 Investigating strategies to prevent the development of WSLs before and throughout fixed orthodontic treatment is a pivotal area of scholarly inquiry. This pertinence arises from the noteworthy incidence rates of WSLs, ranging from 30% to 70%, documented during fixed orthodontic interventions.^3^

 Fluoride-releasing orthodontic adhesives represent a viable approach in the potential mitigation of WSLs.^15^

 Integrating fluoride into orthodontic adhesives presents a practical dilemma concerning fluoride application. Ensuring a sustained supply of fluoride to the enamel surface remains critical even after the composite has fully released its fluoride content. Consequently, recharging the adhesive with sodium fluoride becomes essential to augment the availability of fluoride ions within the medium over a designated timeframe.

 The primary objective of this study was to evaluate the fluoride release characteristics of Vega Ortho UV light-cured orthodontic adhesive at different time intervals and investigate its rechargeability by employing fluoride varnish. Additionally, the study sought to address the uncertainty surrounding the potential impact of these procedures on the SBS of brackets. To achieve this, the investigation involved the assessment of SBS at various time points both before and after the recharge process. Furthermore, the study involved the creation of artificially demineralized lesions on the buccal tooth surface (position of the bracket) to examine the effects of fluoride from adhesive and fluoride varnish on SBS in demineralized tooth surfaces. The research was motivated by the significant clinical concern in orthodontics regarding WSLs observed in patients with fixed orthodontic appliances due to plaque accumulation around orthodontic brackets, necessitating a focused approach on demineralized teeth to address this issue.

 Across many investigations, the SBS exhibited by brackets on intact enamel surfaces was notably higher than the corresponding SBS values observed on demineralized enamel surfaces.^7,16,17^

 Numerous studies have reported that the diminished SBS of brackets on demineralized enamel can be attributed to the presence of atypical enamel surfaces and the absence of resin tag formation. These factors are pivotal in establishing micromechanical interlocking between the adhesive material and the enamel surface.^18,19^

 The fluoride release pattern observed in the groups before recharge exhibited a notable surge in fluoride ion release on the initial day. This can be attributed to the fluoride ions in the adhesive that had not yet been absorbed by the tooth surface. Subsequently, there was a decline in fluoride ion release after one week, followed by a slight increment after one month. These findings coincide with the outcomes reported in prior studies.^5,20^

 The second part of this study was to measure fluoride ion uptake and re-release from orthodontic adhesives after topical fluoride varnish application. This study showed that orthodontic adhesives could take up and re-release fluoride ions after exposure to topical fluoride. Among the after-recharge groups, the one-month group had the highest mean value, possibly because the adhesive released a large amount of fluoride with a large amount of reuptake during recharge, followed by a more significant release. Other groups distributed between these two groups showed a significant difference when compared to each other by the Duncan test.

 The initial surge in fluoride ion release after the topical application of fluoride can likely be linked to the expulsion of fluoride ions retained on the surface or within the pores of the materials during the re-fluoridation process. These observations suggest that the adhesive under investigation can undergo recharging with fluorides introduced through fluoride application techniques.^21^

 The initial rapid release of fluoride ions during the early days following adhesive application bears clinical significance due to its prompt formation of calcium fluoride on the enamel surface when exposed to the oral cavity environment. This occurrence may potentially play a protective role by promoting the remineralization of etched enamel. Moreover, the burst effect of fluoride ion release might confer certain advantageous biological properties, such as bactericidal and/or bacteriostatic effects, immediately after bracket placement. Despite being comparatively weaker in magnitude, even a sub-parts-per-million (sub-ppm) level of fluoride ion release can considerably influence the demineralization/remineralization process, provided the release is sustained over time.^20^

 Nonetheless, prior investigations have yielded varying and conflicting outcomes regarding the impact of fluoride application on SBS. Generally, fluoride pretreatment has been associated with lower SBS values, leading to a diminished bonding efficacy of orthodontic brackets. The topical application of fluoride has been found to interfere with the etching effect of phosphoric acid on enamel surfaces, consequently reducing the bond strength of orthodontic brackets.^7,22-24^

 The present study revealed a positive correlation between fluoride release and SBS, as indicated in Table 4. The SBS values obtained in the month-after groups demonstrated the highest mean value, signifying the maximum benefit of fluoride ions in facilitating the remineralization of the enamel surface during this time frame. This was followed by the SBS values in the week-after group and then in the day-after group, respectively. These results differ from those reported in earlier research, where fluoride-treated groups exhibited lower SBS values. This discrepancy may be attributed to fluoride’s capacity to react with the enamel surface, leading to calcium fluoride and fluorapatite formation, rendering the surface more resistant to demineralization. Notably, in those studies, fluoride application occurred before bracketing, potentially interfering with the etching effect of phosphoric acid on enamel surfaces and consequently reducing the bond strength of dental resin.^18^ In contrast, our investigation implemented fluoride varnish application after the bracketing procedure on demineralized tooth surfaces, which yielded a noteworthy enhancement in the SBS of the orthodontic attachments.

## Conclusion

 The application of fluoride varnish in the vicinity of the orthodontic bracket, bonded with fluoride-containing adhesive, demonstrated a positive effect on the SBS of the bracket to demineralized tooth surfaces. These observations offer valuable insights into the dynamics of fluoride release and its influence on the bonding strength of orthodontic adhesives, providing potential avenues for optimizing orthodontic treatment outcomes.

## Acknowledgments

 The authors thank and appreciate the University of Mosul, the College of Dentistry, for their support of this study.

## Competing Interests

 There were no conflicts of interest regarding this research.

## Ethical Approval

 This study was carried out at the College of Dentistry, University of Mosul, Iraq, in the animal house with the IACUC ethical approval number UM.DENT.2023/3 at 7/3/2023.
